# Impact of Selected Serum Factors on Metastatic Potential of Gastric Cancer Cells

**DOI:** 10.3390/diagnostics12030700

**Published:** 2022-03-12

**Authors:** Marta Tkacz, Maciej Tarnowski, Agata Poniewierska-Baran, Karol Serwin, Anna Madej-Michniewicz, Anna Deskur, Bogusław Czerny, Teresa Starzyńska

**Affiliations:** 1 Department of Physiology, Pomeranian Medical University in Szczecin, 70-111 Szczecin, Poland; maciejt@pum.edu.pl (M.T.); agata.poniewierska@gmail.com (A.P.-B.); 2 Institute of Biology, University of Szczecin, 71-415 Szczecin, Poland; 3 Department of Infectious, Tropical Diseases and Immune Deficiency, Pomeranian Medical University in Szczecin, 71-455 Szczecin, Poland; karolserwin@gmail.com; 4 Department of Gastroenterology, Pomeranian Medical University in Szczecin, 71-252 Szczecin, Poland; kgastro@pum.edu.pl (A.M.-M.); anndes@wp.pl (A.D.); testa@pum.edu.pl (T.S.); 5 Department of Stem Cells and Regenerative Medicine, Institute of Natural Fibres and Medicinal Plants, 62-064 Plewiska, Poland; boguslaw.czerny@pum.edu.pl; 6 Department of General Pharmacology and Pharmacoeconomics, Pomeranian Medical University in Szczecin, 71-210 Szczecin, Poland

**Keywords:** gastric cancer, progression, *HGF*, *SDF-1*, *VEGF*

## Abstract

(1) Background: stromal-derived factor-1 (SDF-1/CXCL12), hepatocyte and vascular-endothelial growth factors (HGF and VEGF) have been shown to facilitate cell motility, proliferation and promote local tumor progression and metastatic spread. Recent research shows the important role of these cytokines in gastric cancer (GC) progression. (2) Methods: 21 gastric cancer patients and 19 healthy controls were included in the study. SDF-1, HGF and VEGF levels were evaluated in sera by ELISA. Patients and control sera were used to stimulate CRL-1739 GC cell line, and chemotaxis, adhesion and proliferation potential were assessed. (3) Results: Concentrations of SDF-1, HGF and VEGF were significantly higher in patients than in controls. Chemotaxis and adhesion assays revealed a significant response of GC cells to patients’ serum. Furthermore, significant relationships were seen between chemotactic/adhesion response and tumor stage. Serum from intestinal early GC patients produced significantly stronger chemotactic response when compared to patients with metastatic spread. In turn, serum from patients with distal metastases significantly increased the adhesion of GC cells when compared to sera from the patients with no distal metastases. We also observed that HGF strongly stimulated the proliferation of CRL-1739 cells. (4) Conclusions: We observed that the sera from GC patients, but also SDF-1, HGF and VEGF used alone, have a strong pro-metastatic effect on CRL-1739 cells. We also demonstrated that the concentration of these cytokines is specifically elevated in the sera of patients in an early stage of malignancy. Our results indicate that SDF-1, HGF and VEGF are very important molecules involved in gastric cancer progression.

## 1. Introduction

Despite recent progress in diagnostic techniques, gastric cancer (GC) remains a clinical problem and a challenge for clinicians and scientists. It is the second most common cancer-related cause of death in the world. In 2018, gastric cancer accounted for 782,685 of the deaths that occurred globally [1]. In Europe and the USA, the 5-year survival rate is only 20–30 percent. Surgery is the main treatment, often followed by chemo/immunotherapy [2,3,4,5,6]. Previous studies indicated that the development and progression of GC is a complex process that involves a variety of cross-linked factors, such as environmental background, genetics, cellular dysfunction, angiogenesis, invasion and metastasis [7]. It was also demonstrated that even in the very early stage of GC, there is a risk of metastatic spread. Thus, in lesions limited to the mucosa, approximately 2% of patients will develop metastases to regional lymph nodes [8]. The mechanisms underlying the multiple processes involved in metastasis development remain unclear [9]. The migration of cancer cells is regulated through multiple chemokine–receptor axes. Factors like SDF-1 (stromal derived factor-1), HGF (hepatocyte growth factor/scatter factor), VEGF (vascular/endothelial growth factor) secreted by stromal cells or tumor-associated macrophages (TAM) attract GC cells through the CXCR4, CXCR7, c-Met and KDR receptors [10,11]. Cells migrate through the blood and lymph vessels, most often into the peritoneum, lymph nodes and liver [12,13,14]. GC cells are characterized by an unusual ability to incite the cooperation with other cell types, like fibroblasts, monocytes, endothelial progenitor cells and endothelial cells. This cooperation, instead of protecting from the pathological phenotype, leads to growth promotion, angiogenesis and migration [10,15,16,17]. This process involves the interaction of several factors—including VEGF, tumor necrosis factor alpha (TNF-α), HGF, epidermal growth factor (EGF) and matrix metalloproteinases (MMPs)—released by both the recruited cells and GC cells. The existence of chronic inflammation, induced by H. pylori infection, contributes to the release of inflammatory mediators, cytokines and growth factors and the promotion of epithelial–mesenchymal transition (EMT) [18]. SDF-1 (also known as CXCL12) is a cytokine that belongs to the chemokine family, secreted by endothelial cells and fibroblasts. SDF-1 affects cell motility, apoptosis, angiogenesis and carcinogenesis through CXCR4 and CXCR7 receptors, and of which expression was observed in several cancers, including GC [19]. Numerous studies indicate that CXCR4/SDF-1 axes play an important role in the survival of cells, their proliferation, migration and adhesion [19,20]. HGF is another multifunctional cytokine. The biological activity of HGF plays a special role in angiogenesis, the adhesion and motility of tumor cells, invasion and the formation of metastases. In the gastrointestinal tract, HGF stimulates the proliferation and migration of intestinal epithelial cells [21,22,23]. Secreted by all cells of mesenchymal origin, including fibroblasts, it has a significant impact on the migration of tumor cells [22,23]. VEGF is strongly associated with angiogenesis. The loss of a single VEGF allele prevents the formation of blood vessels, resulting in the death of the mice at the stage of the embryo [24]. It affects the permeability of blood vessels and the neovascularization in the tumor mass, which considerably improves the microenvironment conditions inside tumor tissue [25,26,27]. As a vascular permeability factor (VPF), it is considered an important element of the occurrence of vascular exudate and the danger of ascites [26,28]. Recent studies have shown that the serum concentration of SDF-1, HGF, and VEGF is elevated in patients with several types of cancers, including breast, liver or ovarian cancer [29,30,31], and what is important is that it was shown that GC tumor mass secretes VEGF and it may serve as biomarker for disease progression and remission [32,33]. 

In the present study, we report that the serum from GC patients, SDF-1, HGF and VEGF, have pro-metastatic effects on the GC CRL-1739 cell line. The concentration of these cytokines is elevated in GC patients from an early stage of malignancy. Our results indicate that SDF-1, HGF and VEGF may promote gastric cancer progression.

## 2. Materials and Methods

### 2.1. Study Design and Description of Patient Cohort: Inclusion and Exclusion Criteria

A total of 21 gastric cancer patients were included in the study. All cases had adequate clinical and pathologic information. At the time of inclusion, none of the patients was undergoing chemotherapy, had received any cytotoxic agents/drugs within the last 12 months before the study nor presented signs of an active infectious disease. The histological type and the stage of GC were assessed by routine histopathologic examination of material obtained after endoscopic or surgical treatment and by the results of computed tomography of the abdomen and chest. Histological types were classified according to the Lauren classification [34]. TNM and UICC (International Union Against Cancer) classifications were used to evaluate the cancer stage. Additionally, cancers were classified as early or advanced according to the criteria of the Japanese Research Society for Gastric Cancer [35]. The clinical data of the GC patients are summarized in Table 1. The GC patients and controls were enrolled at the Department of Gastroenterology, Pomeranian Medical University, Szczecin. The control subjects had no history of cancer. The study was approved by the Bioethics Committee of Pomeranian Medical University (KB-0080/86/09, 18.05.2009).

### 2.2. Collection and Preservation of Serum Samples

Peripheral blood samples were collected before surgery or chemotherapy. The blood samples were centrifuged at 1800 rpm for 15 min at 4 °C, and the serum was transferred to tubes and stored at −80 °C until the time of analysis. 

### 2.3. Cells and Culture Conditions

We used human gastric adenocarcinoma cell line CRL-1739 (ATCC). Gastric cancer cells used for experiments were cultured in Dulbecco’s Modified Eagle’s Medium (Sigma, St. Louis, MO, USA), supplemented with 100 IU/mL penicillin and 10 µg/mL streptomycin (Life Technologies, Inc., Grand Island, NY, USA) in the presence of 10% heat-inactivated fetal bovine serum (FBS, Life Technologies, Waltham, MA, USA). The cells were cultured in a humidified atmosphere at 37 °C in 5% CO_2_ at an initial cell density of 2.5 × 10^4^ cells/flask (Corning, Cambridge, MA, USA), and the medium was changed every 48 h.

### 2.4. Enzyme-Linked Immunoassay

The preoperative serum SDF-1, HGF and VEGF levels were determined by a SDF-1, HGF or VEGF enzyme-linked immunosorbent assay (R&D Systems, Minneapolis, MN, USA) according to the manufacturer’s instructions.

### 2.5. Online Databases and Analysis

Three GEO databases (ncbi.nlm.nih.gov/geo/) (accessed on 16 February 2022) including human gastric cancer specimens and normal tissue were used. GSE13911, GSE19826 and GSE54129 microarray databases were based on GPL570 platform ((HG-U133_Plus_2) Affymetrix Human Genome U133 Plus 2.0 Array). The databases contained a total number of 228 specimens (161 cancer and 67 normal tissue). The genetic expression of SDF-1, HGF and VEGFA was analyzed with the use of the GEO2R online analysis tool (ncbi.nlm.nih.gov/geo/geo2r) (accessed on 16 February 2022). Survival analysis was performed with use of the Kaplan–Meier plotter [36], an online tool that can assess the effect of more than 30,000 genes on survival in 21 tumor types. The survival analysis was based on the mRNA gene chip gastric cancer database containing 875 samples. The hazard ratio (HR) with 95% confidence intervals (CIs) and log-rank *p*-values were calculated. 

### 2.6. Chemotaxis Assay

Polycarbonate membranes (8 µm) were covered with 50 µL of 0.5% gelatin. The cells were detached with 0.5 mmol/L ethylenediaminetetraacetic acid (EDTA), washed in PBS, resuspended in DMEM with 0.5% BSA and seeded at a density of 3 × 10^4^ in 120 µL into the upper chambers of Transwell inserts (Costar Transwell; Corning Costar, Corning, NY, USA). The lower chambers were filled with SDF-1α (300 ng/mL), HGF (10 ng/mL), VEGF-A (10 ng/mL), 1% serum from 21 gastric cancer patients, 1% serum from 19 healthy controls or 0.5% BSA in DMEM (negative control). The concentration of SDF-1, HGF and VEGF were chosen on the basis of dose-response experiments (data not shown) and are higher than physiological. The doses were estimated based on a dose-response assay. After 24 h, the inserts were removed from the Transwell supports. The cells remaining in the upper chambers were stained by HEMA 3 according to the manufacturer’s instructions (Fisher Scientific, Pittsburgh, PA, USA) and were scraped off with cotton wool, and cells that had transmigrated were counted on the lower side of the membrane. 

### 2.7. Cell Adhesion

The cells were resuspended in DMEM with 0.5% BSA. After 4 h, the cells were divided at a concentration of 2.5 × 10^4^ in 100 µL and seeded into 96-well fibronectin-coated plates. The cells were stimulated with optimal doses of SDF-1α (300 ng/mL), HGF (10 ng/mL), VEGF-A (10 ng/mL), 1% serum from 21 gastric cancer patients (1%) and 1% serum from 19 healthy controls for 5 min at 37 °C. Afterwards, the stimulation cells were washed twice in DMEM with 0.5% BSA and counted under a microscope. 

### 2.8. Cell Proliferation

The cells were plated in culture flasks at an initial density of 5 × 10^3^ cells/well in the presence or absence of SDF-1α (300 ng/mL), HGF (10 ng/mL), VEGF-A (10 ng/mL), 1% serum from 2 gastric cancer patients, 1% serum from healthy controls and 1% FBS. The cell number was calculated at 24, 48 and 72 h after culture initiation. At the indicated time points, the cells were harvested from the culture flasks by trypsinization, and the number of cells was determined using a Navios flow cytometer (Beckman Coulter). 

### 2.9. Statistical Analysis

All results are presented as the mean ± standard error of the mean (SEM). Statistical analysis of the data was performed using the unpaired Student’s-*t* test, with *p* < 0.05 considered significant. Statistical comparisons were performed with Fisher’s exact and X^2^ test for nominal variables, as needed. The Mann–Whitney U test was used to analyze the continuous variables (cytokines level and age at diagnosis). IQR: interquartile range. Statistical calculations and visualization were made with commercial software (Statistica v. 13. Statasoft, Warsaw, Poland) and GraphPad Prism 9.3.1 (471). 

## 3. Results

### 3.1. Detailed Patient Characteristics

There were 11 males and 10 females, with a mean age of 64.8 years (range, 46–84 years) in the cancer group and 19 cancer-free, healthy controls (10 males and 9 females, mean age of 59.6 years, range 51–80 years). The cancer group consisted of 10 intestinal and 11 diffuse GC patients. According to the Tumor Node Metastasis (TNM) classification, 5 patients had stage I gastric cancer, 3 had stage II, and 12 patients presented with metastasis (stage IV). Histological analysis revealed “intestinal” type gastric cancer in 10 patients and “diffuse” type in 11 patients. Among the patients with adenocarcinoma, 3 patients showed low-grade I stage tumors and 4 patients showed a moderate/high grade of malignant potential (stage II and III).

### 3.2. High Level of SDF-1, HGF and VEGF in Gastric Cancer Patients’ Serum

The serum SDF-1, HGF and VEGF-A levels were analyzed in 21 GC patients and 19 healthy controls (Table 1). 

**Figure 1:**
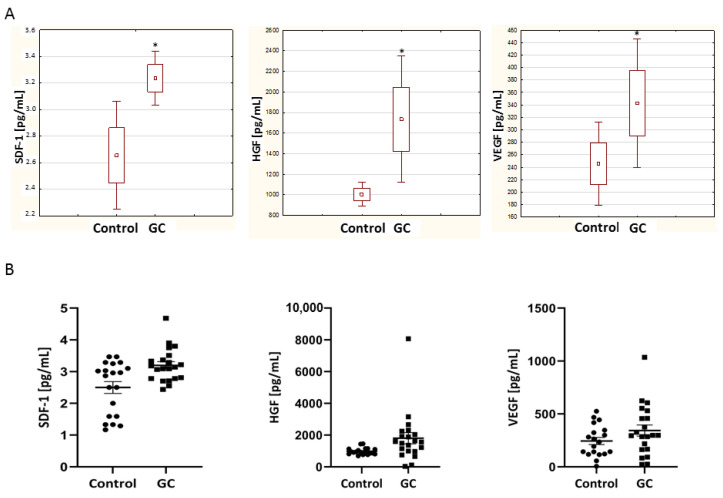
(**A**) The average of SDF-1, HGF and VEGF-A concentrations in gastric cancer and healthy control sera. (**B**) SDF-1, HGF and VEGF-A concentrations in gastric cancer (GC) and healthy control sera. Error bars represent mean ± SEM. * *p* < 0.05.

The distribution of serum SDF-1, HGF and VEGF-A levels in GC patients and normal individuals is shown in Figure 1A,B and Table 1. The average level of SDF-1, HGF and VEGF-A in the GC patients’ serum was 3.2 (SEM 0.11), 1808.59 (SEM 356.38) and 345.59 (SEM 52.54), respectively (Figure 1). The average levels of SDF-1, HGF and VEGF in the serum from controls was 2.66 (SEM 0.20), 1003.74 (SEM 46.78) and 245.6 (SEM 32.22), respectively (Figure 1). The concentration of SDF-1, HGF and VEGF in the GC patients was significantly higher than in the healthy controls (Figure 1). 

**Figure 2:**
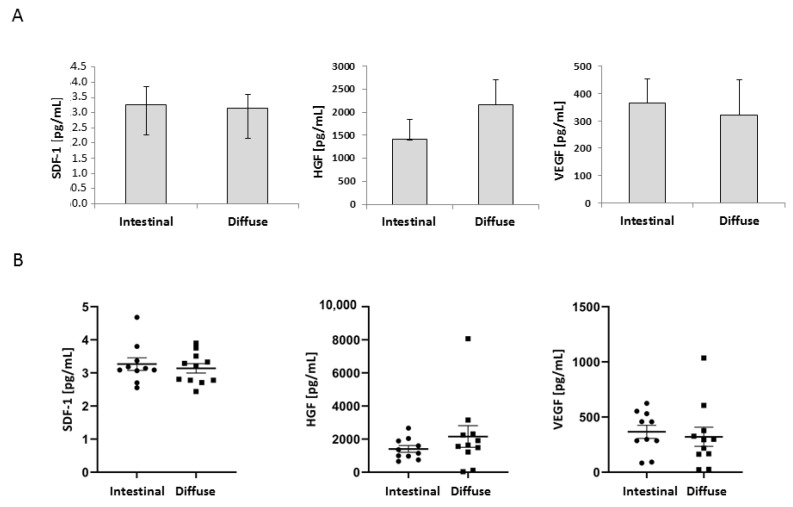
(**A**) Concentration of SDF-1, HGF and VEGF-A in the intestinal and diffuse type of gastric cancer. (**B**) Individual concentration of SDF-1, HGF and VEGF-A in the intestinal and diffuse type of gastric cancer. Error bars represent mean ± SEM.

**Figure 3:**
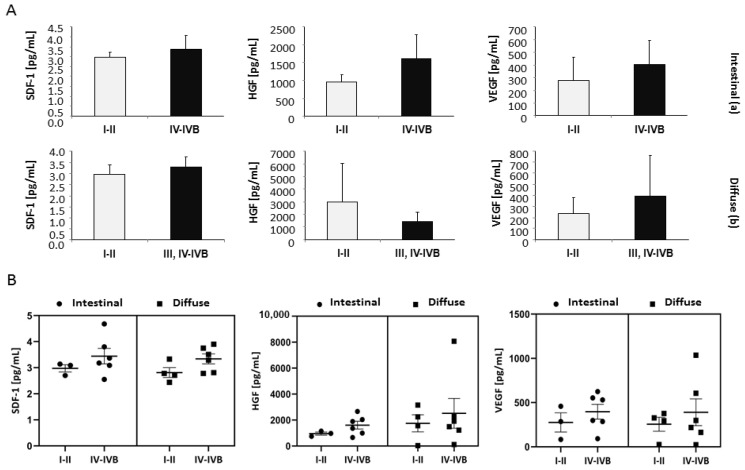
(**A**) Concentration of SDF-1, HGF and VEGF depending on the stage in intestinal (**a**) and diffuse (**b**) gastric cancer. (**B**) Individual concentrations of SDF-1, HGF and VEGF depending on the stage in intestinal (I-II and IV-IVB) and diffuse (I-II and IV-IVB) gastric cancer. Error bars represent mean ± SEM.

There was no significant correlation between serum SDF-1, HGF, VEGF-A levels, GC histology and stage (Figure 2A,B and Figure 3A,B). Further analysis showed statistical significance between healthy control serum HGF levels and intestinal IV-IVB (*p* = 0.03), diffuse III, IV-IVB (*p* = 0.01) and total diffuse (*p* = 0.006) gastric cancer. The statistical relevance was also shown for age at diagnosis (median years) and intestinal IA-IB patients (*p* = 0.01), data shown in Table 2.

**Figure 4:**
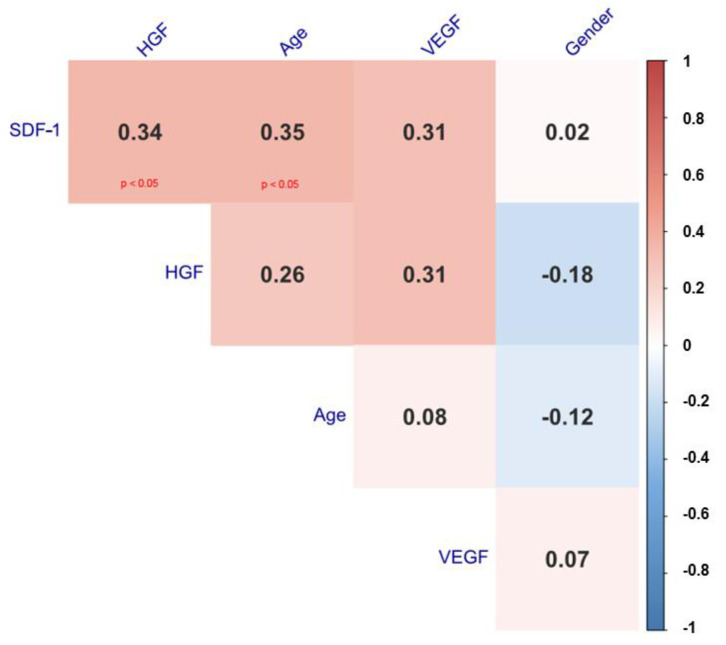
Spearman correlation heat map with correlation coefficient and significance levels.

In addition, we found a strong positive correlation between SDF-1 vs. HGF (0.34, *p* < 0.05), SDF-1 vs. Age (0.35, *p* < 0.05), SDF-1 vs. VEGF-A (0.31), HGF vs. Age (0.26) and HGF vs. VEGF-A (0.31), which are shown in Figure 4. Due to the small number of correlations, there was no basis for creating more advanced regression models.

### 3.3. High Expression of SDF-1, HGF and VEGF-A in Tumor Tissues Decreases Gastric Cancer Patients’ Overall Survival

In the bioinformatics study, three (GSE13911, GSE19826 and GSE54129) microarray databases were screened for differences in expression between GC and normal specimens. The analysis indicated a relatively uniform increase in the genetic expression of SDF-1, HGF and VEGFA in GC samples when compared to normal adjacent tissue; however, these differences did not reach statistical significance (set to *p* < 0.05) in GSE13911 or GSE19826. Interestingly, when we used the GSE54129 database, which included 111 GC samples and normal gastric mucosa from 21 volunteers who underwent gastroscopy for health examination, it was noted that SDF-1 (probe ID: 203666_at) and HGF (probe ID: 210998_s_at) were significantly upregulated (logFC = 1.324 and 0.998, respectively; *p* < 0.05). VEGF-A expression was also significantly different, but the change (logFC = 0.439 for probe ID: 210513_s_at) was minimal. 

**Figure 5:**
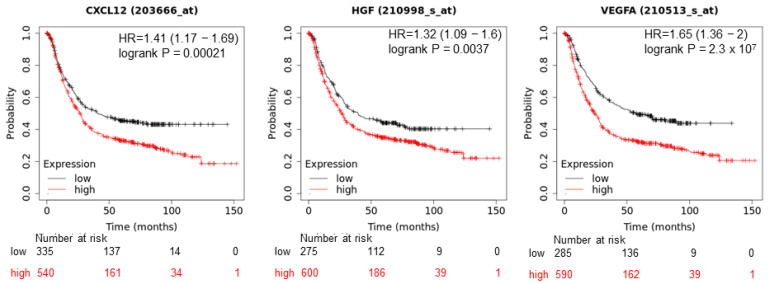
Kaplan–Meier overall survival analyses of patients with gastric cancer based on expression of SDF-1/CXCL12, HGF and VEGFA.

Overall survival analysis of the selected genes was performed using the Kaplan–Meier plotter. The hazard ratio (HR) with 95% confidence intervals (CIs) and log-rank *p*-values were calculated. The analysis showed a decrease of overall survival and indicated poor prognosis when SDF-1, HGF and VEGF-A were highly expressed in tumor tissues (Figure 5).

### 3.4. SDF-1, HGF, VEGF-A and Serum from Gastric Cancer Patients Promote Migration of Gastric Cancer Cells

**Figure 6:**
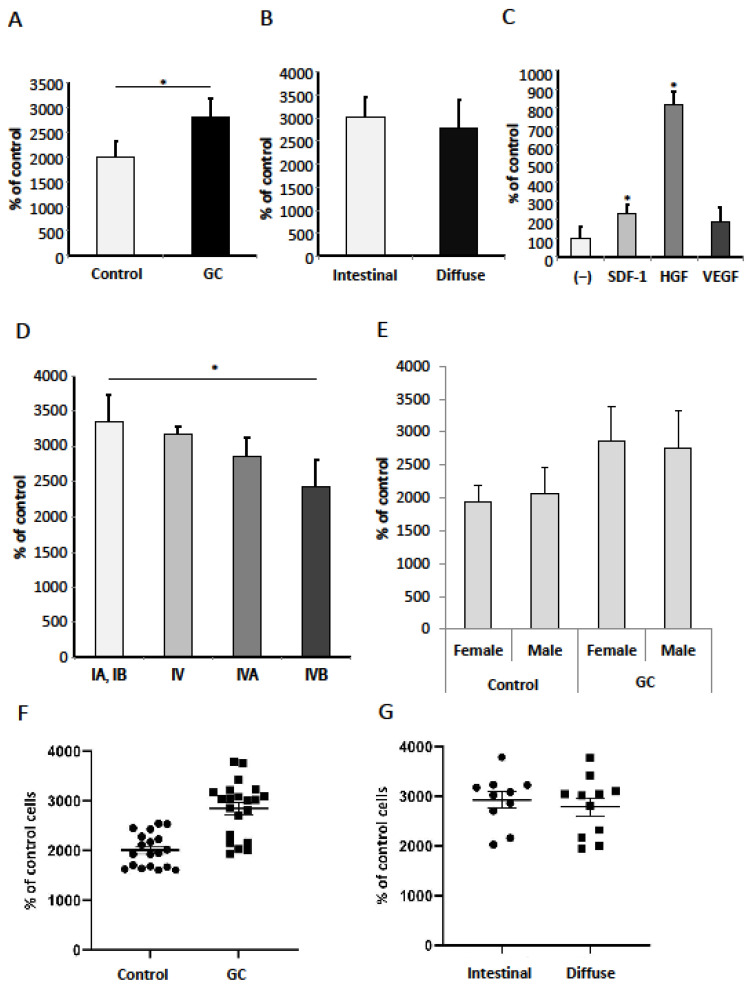
Chemotactic response of gastric cancer cell line CRL-1739 (**A**) to gastric cancer (GC) and healthy control sera; (**B**) to 1% serum depending on the type of gastric cancer according to the Lauren classification; (**C**) in the gradient of SDF-1 (300 ng/mL), HGF (10 ng/mL) and VEGF (10 ng/mL); (**D**) depending on the stage in the intestinal gastric cancer; (**E**) in male and female gastric cancer patients and healthy controls. (**F**) Individual responses of gastric cancer cells to healthy control and gastric cancer (GC) sera. (**G**) Individual responses of gastric cancer cells with regard to the type of gastric cancer. All experiments were repeated three times with similar results. Error bars represent mean ± SEM. * *p* < 0.05.

SDF-1, HGF and VEGF have been reported to promote the directional migration and invasion of human cancer cells [19,37,38]. First, we checked the chemotactic potential of the serum from GC patients. We noted that the serum from GC patients significantly increased the chemotactic response of CRL-1739 cells when compared to healthy controls (Figure 6A). Next, we examined the migratory activity of gastric cancer cells in the intestinal and diffuse type of stomach cancer group. No significant difference was observed between the intestinal and diffuse type of GC (Figure 6B). We also observed that SDF-1 and HGF significantly increased the chemotactic potential of CRL-1739 cells (Figure 6C). Further analysis of the clinical data showed a significantly increased chemotactic response in patients with early cancer (I) when compared to sera obtained from patients with metastatic spread (IVB). This phenomenon was only observed in the intestinal type (Figure 6D). There was no statistical significance when the diffuse type of gastric cancer was analyzed (data not shown). Additionally, there was a higher (but not statistically significant) response in females than males in the gastric cancer group, and this relation was not observed in the control group (Figure 6E). An evaluation of the chemotactic response with relation to age showed similar values in both groups (GC patients and healthy controls) (data not shown).

### 3.5. SDF-1, HGF, VEGF-A and Serum from Gastric Cancer Patients Promote Adhesion of Gastric Cancer Cells

**Figure 7:**
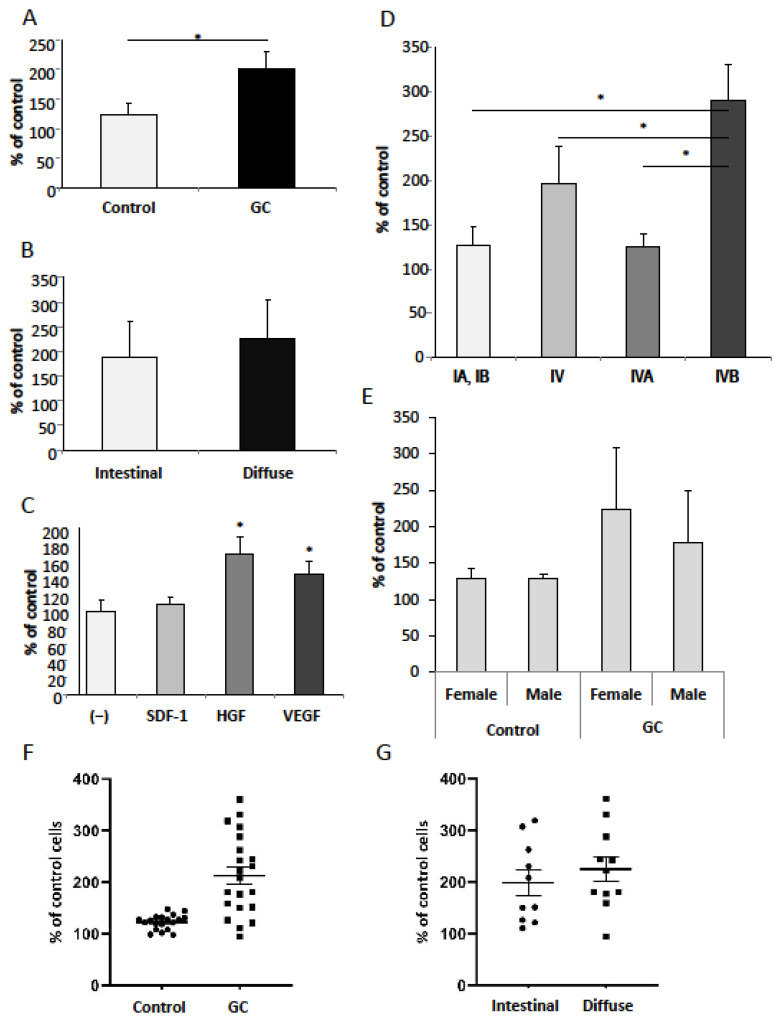
(**A**) Adhesion of gastric cancer cell line CRL-1739 to fibronectin after 5 min stimulation with 1% gastric cancer (GC) and healthy control sera; (**B**) depending on the type of gastric cancer according to Lauren classification; (**C**) in the presence of SDF-1 (300 ng/mL), HGF (10 ng/mL) and VEGF (10 ng/mL); (**D**) depending on the stage in the intestinal gastric cancer; (**E**) in male and female gastric cancer patients and healthy controls. (**F**) Individual adhesion of gastric cancer cells to healthy control and gastric cancer (GC) serum. (**G**) Individual adhesion of gastric cancer cells depending on the type of gastric cancer. All experiments were repeated three times with similar results. Error bars represent mean ± SEM. * *p* < 0.05.

As shown in Figure 7A, the adhesion of GC cells was significantly increased in GC patients when compared to the healthy controls. Next, we noted a significantly increased adhesive response in the presence of HGF and VEGF (Figure 7C). No significant difference in the adhesive response was observed between the intestinal and diffuse type of GC (Figure 7B), but it was dependent on the tumor stage (Figure 7D). The analysis of the clinical data showed higher adhesive response to the serum obtained from the patients with distal metastases (IVB) when compared to sera from the patients with no distal metastases (I and IVA). This phenomenon was only observed in serum from patients with intestinal-type GC. Further analysis demonstrated that sera derived from GC females increased the adhesion ability of CRL-1739 cells when compared to the GC male sera, and this relation was not observed in the control group (Figure 7E). An evaluation of the adhesion with relation to age showed similar values in both groups (GC patients and controls) (data not shown).

### 3.6. SDF-1, HGF, VEGF-A and Serum from Gastric Cancer Patients Promote Proliferation of Gastric Cancer Cells

**Figure 8:**
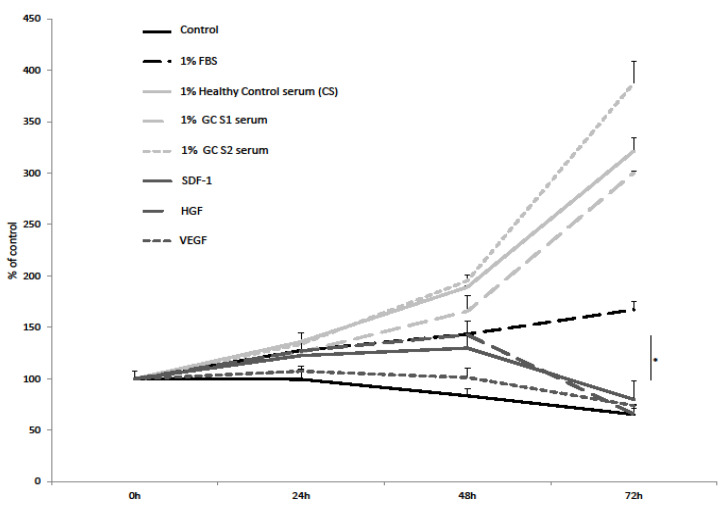
Proliferation of gastric cancer cell line CRL-1739 in gastric cancer and control serum. CRL-1739 cells were stimulated with: 1% of gastric cancer patient serum (GC S1, GC S2), 1% of healthy control serum (CS), SDF-1 (300 ng/mL), HGF (10 ng/mL), VEGF (10 ng/mL), 1% of fetal bovine serum (FBS) and 0.5% BSA (control). The experiment was repeated three times with similar results. * *p* < 0.05.

The proliferation assay showed that SDF-1 and HGF stimulate the proliferation of CRL-1739 cells (Figure 8). VEGF had no effect on GC cells’ proliferation. Interestingly, HGF stimulated the cellular activity as strongly as 1% FBS at 48 h. Analysis demonstrated that, at 48 h, HGF alone significantly improves cell survival when compared to VEGF alone. The growth of the gastric cancer cell line was strongly stimulated by 1% human serum, and the effect was stronger than 1% FBS. There was no significant difference between the proliferation triggered by the GC serum versus the healthy control serum (Figure 8).

## 4. Discussion

In this study, we demonstrated that the SDF-1, HGF and VEGF-A are elevated in GC patient serum and CG CRL-1739 cells characterize with significant chemotactic and adhesive responses. Interestingly, the levels of these cytokines are increased from an early stage of malignancy, including tumors invading mucosa, in both intestinal and diffuse types. The comparison of the activity of the serum from GC patients and controls showed a significantly higher chemotactic response and cell adhesion. An interesting observation from our study is the significant decrease of GC cells’ chemotactic response with increasing tumor stage. Thus, sera from patients with early intestinal GC, when compared to sera from patients with metastatic spread (IVB), was the strongest chemotactic attractant for GC CRL-1739 cells. In contrast, in diffuse gastric cancer, the lowest chemotactic response was observed in the early stage. These observations should be confirmed on a bigger study group; however, it seems that this finding is not accidental. The differences may result from the distinct etiology of both histological types and the mechanisms of their formation, as well as the cell biology. This may indicate an important role of SDF1/HGF/VEGF in early intestinal gastric cancer spread. What is more, with the use of GEO database analyses and Kaplan–Meier plotting, we noted that the high expression of the three evaluated factors is related to a decreased overall survival of GC patients.

Many studies have shown that the axis of SDF-1/CXCR4 plays a crucial role in the peritoneal and lymph node metastasis of GC [19,38,39,40]. The peritoneum is a common metastatic site among patients with GC and often its consequence is lethal. The direction of migration is not accidental, as peritoneal mesothelial cells abundantly secrete chemokine SDF-1 [38,39,40]. Our data confirmed the key role of the SDF-1/CXCR4 axis in the motility of gastric cancer cells. Despite a number of in vitro studies confirming the role of SDF-1 in gastric cancer cell migration, there are still doubts about the clinical relevance of these observations. These doubts arise from immunohistological results showing no clinical significance of CXCR4 expression [39,41]. SDF-1 has no significant effect on GC adherence and has a primary role in initiating growth, migration and metastasis. This corresponds to a study by Iwasa et al., in which the SDF-1/CXCR4 axis has been shown to be important in GC cells’ proliferation in the auto- and paracrine mechanism [38]. Other studies demonstrated the positive effect of the chemokine SDF-1 in the process of the proliferation of gastric cancer cell line NUGC4, characterized by the high expression of CXCR4 [28].

HGF is one of the better-known factors in gastric cancer biology. HGF in the gastrointestinal tract is responsible for the modulation of cell proliferation, as well as the migration of intestinal epithelial cells, and may, therefore, directly influence the affected tissue, promoting processes associated with rapid tumor growth [21,22,23]. The overexpression of c-Met occurs in 40–80% of gastric cancer patients [42,43]. Wang et al. demonstrated that the high expression level of HGF and c-MET were indicators of unfavorable clinical outcomes in gastric cancer [44]. Thus, the expression of HGF was associated with an invasive and/or metastatic phenotype of gastric cancer cells. In our data, we observed that HGF strongly stimulated chemotaxis and adhesion. Moreover, we found that HGF, similarly to bovine serum, did affect the proliferation of GC cells, confirming an important role of HGF in the growth of gastric cancer. Thus, the use of a c-Met kinase inhibitor (e.g., foretinib and tivantinib) in the treatment of gastric cancer may be worth considering [45].

Tumor angiogenesis and lymphangiogenesis play an essential role in the growth, invasion and metastatic spread of solid neoplasms by facilitating the delivery of oxygen, nutrients, and growth factors to tumor cells [33]. VEGF is one of the most important factors in this process. In our study, VEGF strongly increased the adhesion of GC cells, but there was no significant effect on the gastric cancer cells’ migration and proliferation. These results may indicate an important role of VEGF in the process of the colonization of premetastatic niches as a consequence of VEGF secretion, for example by epithelial cells. Prager et al. reported that rapamycin significantly reduced the chemotactic response of gastric cancer cells (MKN-45) in a gradient of VEGF, but did not significantly affect the level of proliferation [46]. In turn, bevacizumab, used in the treatment of advanced gastric cancer, blocked the migration of cells [42]. Increased vascular permeability due to VEGF is considered an important driver of increased ascites production in gastric cancer [47]. 

The analysis of cells’ adhesion showed results converse to chemotaxis. The adhesive potential was increased in the serum from the patients with distal metastases (IVB) in intestinal gastric cancer, which is the opposite of a chemotactic response. This interesting observation may result from the gastric cancer cells’ biology, their strong motility and their spread potential. We conclude that HGF may be one of the most important factors regulating gastric cancer metastasis. Not only does it promote cell migration and adhesion, but it also plays an important role in gastric adenocarcinoma proliferation and survival. Gender differences in incidence, mortality, overall survival and cancer-specific survival have been observed in several types of cancer [48]. In our study, female serum increased the adhesion of GC cells, which may affect a different process and prognosis in gastric cancer. 

It should be emphasized that the observed significant chemotactic, adhesive and proliferative serum potential is an additional effect of SDF-1, HGF, VEGF and also other substances participating in the regulation of the metastasis process. The chemotaxis of cancer cells is dependent on the tumor microenvironment and is a primary element to determine metastatic spread [49]. 

In conclusion, SDF-1, HGF and VEGF may promote gastric cancer progression. Cytokines and serum from patients with GC, including early cancer, have strong chemotactic properties that promote the migration, adhesion and proliferation of GC cells. However, this study has some important limitations that refer to the number of patients included in the study and the single cell line used herein. The replication of this research on a larger cohort of patients is certainly needed to improve the applicability of the results in diagnostics and clinical practice.

## Figures and Tables

**Table 1:** Characteristics of gastric cancer patients and healthy controls.

Clinicopathological Parameters	Cases (n = 21)	Healthy Controls (n = 19)
Age (years)	64.8 (3.15) ^1^	59.6 (1.88) ^1^
Gender		
− Male	11 (52.4%)	10 (52.6%)
− Female	10 (47.6%)	9 (47.4%)
Tumor histology		
− Intestinal	10 (47.6%)	
− Diffuse	11 (52.4%)	
TNM stage		
− IA	3 (14.3%)	
− IB	2 (9.5%)	
− II	3 (14.3%)	
− III	1 (4.8%)	
− IV	4 (19.0%)	
− IVA	2 (9.5%)	
− IVB	6 (28.6%)	
Serum levels (pg/mL)		
− SDF-1	3.2 (0.11) ^1^	2.66 (0.20) ^1^
− HGF	1808.59 (356.38) ^1^	1003.74 (46.78) ^1^
− VEGF-A	345.59 (52.54) ^1^	245.6 (32.22) ^1^

^1^ Standard error of the mean (SEM).

**Table 2:** Characteristics of clinicopathological groups compared to healthy controls. * *p* < 0.05.

	Control	*p*	Intestinal (IA and IB)	*p*	Intestinal (IV-IVB)	*p*	Diffuse (IA, IB, II)	*p*	Diffuse (III, IV-IVB)	*p*	Total Intestinal	*p*	Total Diffuse	*p*
	n = 19		n = 3		n = 7		n = 4		n = 7		n= 10		n= 11	
Male, n (%)	10 (52.63)	ref	1 (33.33)	0.53	5 (71.43)	0.39	1 (25.00)	0.31	3 (42.86)	0.66	6 (60.00)	0.7	4 (36.36)	0.39
Female, n (%)	9 (47.37)		2 (66.67)		2 (28.57)		3 (75.00)		4 (57.14)		4 (40.00)		7 (63.64)	
Serum level of SDF-1 (pg/mL), median	2.96	ref	3.09	0.7	3.18	0.73	2.75	0.97	3.29	0.06	3.11	0.1	3.22	0.16
(IQR)	(1.59–3.27)		(2.89–3.11)		(3.08–3.59)		(2.64–2.92)		(3.02–3.63)		(3.07–3.32)		(2.78–3.42)	
Serum level of HGF (ng/mL), median	0.92	ref	0.97	1	1.60	0.03 *	1.90	0.13	1.64	0.01 *	1.26	0.08	1.64	0.006 *
(IQR)	(0.82–1.12)		(0.86–1.06)		(1.18–1.96)		(1.18–2.47)		(1.35–2.11)		(0.97–1.82)		(1.35–2.28)	
Serum level of VEGF (pg/mL), median	235.18	ref	285.19	0.85	457.40	0.06	310.50	0.78	218.00	0.6	377.80	0.1	293.90	0.58
(IQR)	(131.88–335.22)		(184.04–371.59)		(291.69–541–750		(226.95–339.45)		(164.85–451–77)		(285.19–512.22)		(164.85–351.80)	
Age at diagnosis, median years	59	ref	73	0.01 *	69	0.39	56	0.49	59	0.69	72	0.06	59	0.97
(IQR)	(54–65)		(72–79)		(53–81)		(49–62)		(52–75)		(59–83)		(49–71)	

## Data Availability

The data presented in this study are available on request from the corresponding author.
